# Anticipatory parental effects in a subtropical lizard in response to experimental warming

**DOI:** 10.1186/s12983-018-0296-3

**Published:** 2018-12-05

**Authors:** Bao-Jun Sun, Yang Wang, Yong Wang, Hong-Liang Lu, Wei-Guo Du

**Affiliations:** 1 Key Laboratory of Animal Ecology and Conservation Biology, Institute of Zoology, Chinese Academy of Sciences, Beijing, 100101 People’s Republic of China; 2 School of Biological Sciences, Hebei Normal University, Shijiazhuang, China; 3 Hangzhou Key Laboratory of Animal Adaptation and Evolution, Hangzhou Normal University, Hangzhou, 310036 China; 4 Center for Excellence in Animal Evolution and Genetics, Chinese Academy of Sciences, Kunming, 650223 China

**Keywords:** Climate change, Growth, Hatchling, Parental effect, Reptile, Survival, Thermal adaptation, Transgenerational phenotypic plasticity

## Abstract

Parental effects may produce adaptive or maladaptive plasticity that either facilitates persistence or increases the extinction risk of species and populations in a changing climate. However, empirical evidence of transgenerational adaptive plastic responses to climate change is still scarce. Here we conducted thermal manipulation experiments with a factorial design in a Chinese lacertid lizard (*Takydromus septentrionalis*) to identify the fitness consequences of parental effects in response to climate warming. Compared to present climate conditions, a simulated warming climate significantly advanced the timing of oviposition, depressed the immune capability of post-partum females, and decreased the hatching success of embryos, but did not affect female reproductive output (clutch size and egg mass). These results indicate that maternal warming negatively affects female health, and embryonic hatchability. More interestingly, we found that offspring from parents exposed to warming environments survived well under a simulated warming climate, but not under a present climate scenario. Accordingly, our study demonstrates anticipatory parental effects in response to a warming climate in an ectothermic vertebrate. However, the fitness consequences of this parental effect will depend on future climate change scenarios.

**Electronic supplementary material:**

The online version of this article (10.1186/s12983-018-0296-3) contains supplementary material, which is available to authorized users.

## Introduction

The global climate change imposes extensive and profound impacts on species, populations and ecosystems [1–3]. In response to changing climate, some species have shifted their distribution to higher latitudes or elevations to track more suitable habitats for their survival and reproduction. Other species have stayed in their current habitats and instead display phenotypic plasticity (e.g., shifts in phenology, physiological acclimation and life-history changes) and/or genetic adaptation in response to climate change [4–8]. Understanding the processes that determine the sensitivity, resilience, and adaptability of a species is critical for predicting its vulnerability to climate change [9–11].

Phenotypic plasticity, which is the ability of a genotype to express diverse phenotypes in different environmental conditions, provides organisms with the capability to alter their phenotypes in response to environmental changes [12, 13]. Phenotypic plasticity is not only a fast process that allows organisms to cope with climate change in the short-term, but also could have long-term effects on species fitness. For example, environmentally induced plasticity could affect offspring fitness through diverse mechanisms such as transgenerational epigenetic inheritance and genetic assimilation [14, 15]. The ‘parental effect’ is a special case of phenotypic plasticity whereby the parental environment substantially affects a diverse array of offspring phenotypes (e.g., morphology, behavior, physiology, and life-history traits), which in turn determine the rapid response of populations to changing environments [16–24] . Parental effects may be adaptive and enable parents to adjust offspring phenotypes (e.g. growth and survival) to local environment changes [25, 26]. Alternatively, parental effects may be neutral or even detrimental (negative physiological side effects) to the survival of species or populations in a changing environment [16]. In either case, parental effects are likely to have far-reaching ecological and evolutionary consequences in different circumstances [27, 28].

The study of the relationship between phenotypic plasticity and global climate change has attracted increasing attention in the last few decades [29–33], while numerous studies have demonstrated substantial plastic responses of physiological and life history traits to spatial and temporal heterogeneity in environmental conditions in a diversity of plants and animals [13, 20, 34–37]. A growing number of theoretical predictions and early empirical evidence suggest that organisms might exhibit adaptive responses to climate change via transgenerational phenotypic plasticity (parental effects) that can mount fast responses, in addition to those intragenerational (fixed developmental or reversable acute) plasticity. This, in turn, suggests that in addition to evolutionary adaptation over longer time scales, plasticity driven by parental effects could be a highly effective mechanism to buffer populations against rapid environmental change [8, 38–40]. The ecological and evolutionary consequences of parental effects with respect to climate change largely depend on the degree to which parental and offspring environments are matched. The majority of previous studies on transgenerational plastic responses induced by climate change aimed to identify the existence of parental effects, but with limited efforts focused on elucidating the fitness consequences of parental effects in different environments [31, 38, 40]. Empirical tests of the adaptive significance of parental effects in the context of climate change requires a fully factorial experimental design that manipulates both parent and offspring environments [18, 22].

Reptiles are excellent models for studies on the adaptive significance of parentally mediated changes in offspring life histories due to climate warming, though such studies are still rare due to logistical difficulties (e.g. manipulation of a warming scenario in large enclosures) (but see [41]). As ectothermic vertebrates, reptiles have intrinsically restricted abilities of long-distance dispersal and are highly dependent upon external environmental conditions and therefore particularly sensitive to climatic change [42, 43]. In addition, environmental temperatures experienced by gravid female reptiles have profound effects on embryonic development and offspring phenotypes [18, 19, 40, 44, 45].

To identify the adaptive significance of parentally mediated changes in offspring life history in response to climate change, we conducted simulated warming experiments (present and future warming climate on parents × present and future warming climate on offspring). We then measured the effect of these warming treatments on female reproductive output and body condition, embryonic development, and offspring phenotypes (locomotor performance, growth and survival) in a lacertid lizard, *Takydromus septentrionalis*. Parents and their offspring would respond to warming climate scenarios in a diversity of ways. For example, warming could benefit parents but not offspring, or benefit offspring but not parents. Here we mainly focus on the following three hypotheses. First, if a warming climate improves growth, reproduction, and survival of lizards as seen in some temperate species [46, 47], then parents and offspring would perform better overall in the warming climate than in the present climate. Second, if a warming climate has negative effects on offspring as shown in some other lizards [42, 43], then parents and offspring would perform worse overall in the warming climate than in the present climate. Third, if parents are capable of adjusting offspring phenotype via predictive adaptive plasticity, offspring would perform better in thermal environments similar to that of their parents, as suggested by the environmental matching hypothesis [25, 48–51].

## Materials and methods

### Study species

The northern grass lizard, *Takydromus septentrionalis*, is a lacertid lizard (up to 80 mm snout-vent length [SVL]), which is widely distributed in central and southern China [52]. In this species, selected body temperature averages 30 °C, while field body temperatures average 28.3 °C in May and 32.4 °C in July for a population from Zhoushan islands, Zhejiang [53]. As an oviparous species, females produce multiple clutches with a clutch size of 1–5 eggs from April to July [54]. As is typical of developing embryos in many oviparous squamates, *T. septentrionalis* embryos are retained in utero for approximately one third of total developmental time, and are almost fully developed (stage 25-26) at oviposition [55, 56]. The gravid period is likely over 20 days and the mean incubation period at 24 °C (roughly the mean temperatures of May and Jun) is approximately 45 days in this species [57]. Thermal conditions experienced by females affect clutch frequency but not clutch size or offspring size [19, 58]. Incubation at high temperatures (> 30 °C) reduces hatching success and produces hatchlings that are smaller in size, and that presents slower locomotor speeds compared to those incubated at lower temperatures [57].

### Experimental design and thermal treatments

**Fig. 1:**
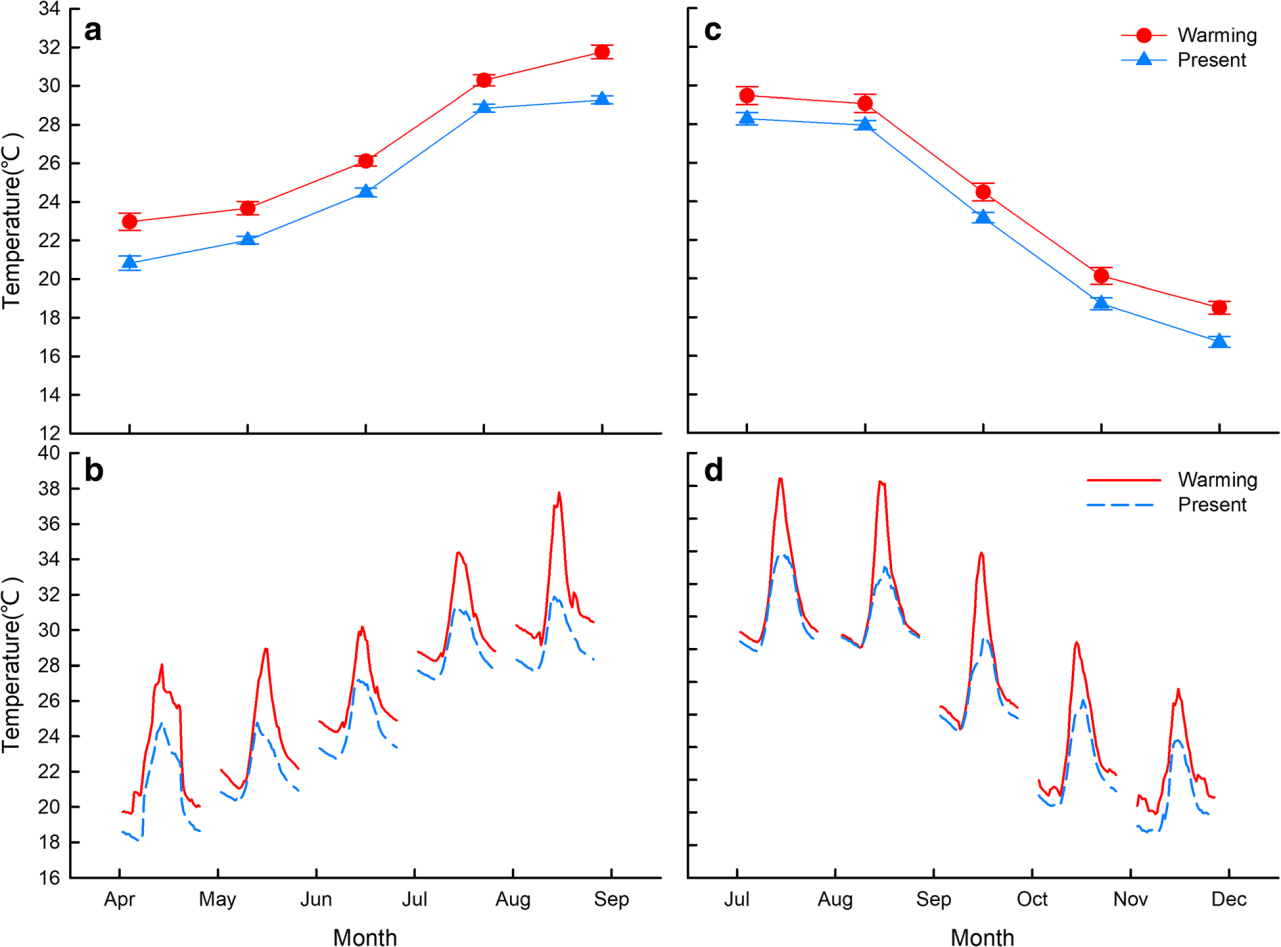
Monthly average (**a**) and daily average (**b**) temperatures experienced by adults (gravid females and males) and monthly (**c**) and daily (**d**) average temperatures experienced by offspring of *Takydromus septentrionalis* in outdoor enclosures. Apr: April; May: May; Jun: June; Jul: July; Aug: August; Sep: September; Oct: October; Nov: November

By the end of the current century, the global mean surface temperature is predicted to increase 0.3–4.8 °C, depending on various global emissions scenarios [59]. We designed a factorial experiment [two parental thermal treatments (Parental warming climate, PWC; Parental present climate, PPC) × two offspring thermal treatments (Offspring warming climate, OWC; Offspring present climate, OPC)] to identify the effect of parental thermal conditions on female reproductive traits, as well as the effect of parental and offspring thermal conditions on offspring traits. The parental and offspring thermal experiments were carried out in eight outdoor enclosures (3 × 3 × 1 m) with natural vegetation, which were built in Hangzhou, Zhejiang (30° 27’ N, 120° 20′ E). Four enclosures were half-covered with a shade net to mimic tree shade in the natural habitat of this species, and used as present climate enclosures to represent the current climate. Another four enclosures were used for the warming climate treatment, over which we suspended two infrared heaters (1000 W, NSB-10TQ13; Xianfeng Instrument Ltd., Zhejiang, China), 1 m above the enclosure ground level. Ambient temperatures within the enclosures were recorded every 30 min using thermochron iButtons (DS1921, MAXIM Integrated Products Ltd., Texas, USA; diameter 15 mm, height 6 mm; *n* = 3 in each enclosure). From April to early November, the average ambient temperature was 1.7 °C or 1.4 °C higher in the warming climate than the present climate treatments for parents (Apr to Aug) or offspring (Jul to Nov) (Fig. 1; see Results for the details of thermal regimes).

### Female collection, reproduction and egg incubation

During the initial stages of reproductive season (From late March to early April), 60 adult *T. septentrionalis* (40 females and 20 males) were captured from Zhoushan islands (30° 09’ N 121° 94′ E) in Zhejiang province of eastern China. This area has an average annual temperature of 16.7 °C and average precipitation is 1222.6 mm per year (http://data.cma.cn/). The collected lizards were transferred to our laboratory in Hangzhou, where the enclosures located. Mean daily temperatures are very similar in the two sites of Zhoushan and Hangzhou during both the breeding season (April–November) and the entire year (see details in Additional file 1: Figure S1). Lizards were individually marked by toe-clip, measured (snout-vent length [SVL] ±1 mm) and weighed (± 0.001 g). We then assigned adults to two present climate and two warming climate enclosures randomly in early April, with 10 females and 5 males in each outdoor enclosure. One female from the present climate treatment escaped during the experiment and was therefore excluded from subsequent analysis. Food (larvae of *Tenebrio molitor* and crickets) and water were provided ad libitum. Active body temperatures of lizards measured on May 1st verified that lizards exposed to the warming climate treatment had higher body temperatures than those from the prewent climate treatment (Additional file 1: Figure S2). Females were checked for gravidity every week, and females with oviductal eggs were then transferred to small containers (200 × 150 × 200 mm) filled with a 2-cm deep substrate of moist sand for oviposition. The females laid their eggs within three days, and each container was checked three times a day for freshly laid eggs. Once found, clutch size was recorded and all eggs were promptly weighed (± 0.001 g). The eggs (*n* = 347) were then half-buried in moist vermiculate (− 220 kPa) [60] inside small boxes (150 × 80 × 50 mm) and incubated at 24 °C. We added water to the substrate regularly to compensate for evaporative loss and water absorbed by eggs. We also moved the boxes among shelves every two days to minimize any effects of thermal gradients inside the incubator (Binder 240, Germany). The postpartum females were measured (±1 mm) and weighed (± 0.001 g) and returned to their original enclosures. The females produced multiple (1–9) clutches of eggs when they were kept in the enclosures during the reproductive season from early April to August. Eggs from early clutches of 39 females before August were used in the incubation experiment, while eggs from later clutches in August were unfertilized and thus excluded from the incubation, but still counted as reproductive output.

### Body condition and cellular immune response of postpartum females

At the end of the reproductive season (August), we measured (±1 mm) and weighed (± 0.001 g) the postpartum females again to evaluate the effects of the two climate treatments on their body condition (using the ratio of body mass to SVL as an index of body condition). Then we assessed their cellular immune response by administering an injection (20 μL) of 50 mg phytohemagglutinin (PHA) into the right foot of postpartum *T. septentrionalis* females (*n* = 30). Thickness of the right and left foot (±1 mm) was measured 24 h after the PHA injection. The difference in thickness between the right and left foot is considered as the index of immune response [61].

### Body size, locomotor performance, growth rate and survival of offspring

After the first hatchling emerged, we checked the boxes three times a day for newly-hatched lizards. We calculated hatching success as the percentage of eggs that hatched successfully, and incubation period as the days from oviposition to hatching. We measured (±1 mm) and weighed (± 0.001 g) each hatchling, and assessed its locomotor performance immediately by running the lizards along a 2-m-long wooden racetrack. To standardize body temperatures of lizards prior to each locomotor trial hatchlings were acclimated in an incubator at 30 °C for 30 min. Locomotor performance of each lizard was recorded with a Panasonic video camera (NV-GS38). Videotapes were then examined to determine measures of sprint speed over the fastest 200-mm interval and average speed over a 500-mm interval.

After the locomotor performance test, we assigned the hatchlings from each clutch as evenly as possible to two present climate and two warming climate enclosures. The hatchlings were kept in the enclosures from late June to November. Food (larvae of *Tenebrio molitor* and crickets) and water were provided ad libitum. Every two weeks after birth, the hatchlings were recaptured from enclosures and re-measured for SVL (±0.01 mm) and re-weighed (±0.001 g). Growth rates of hatchlings were calculated using the growth rate constant K from the Gompertz model, which represented the fastest absolute growth during the experiment [62]. Date of death for each offspring was recorded, and offspring survival (an important indicator of fitness) was assessed over the 5 months from birth.

### Statistical analysis

All analyses were conducted using SPSS Statistics software (v22; IBM Corporation, 2014). Data were normalized by log-transformation when necessary. The difference in ambient temperature between the present climate and warming climate thermal treatments was tested using generalized linear mixed models, enclosure ID as the random effects. The effect of parental thermal treatment on female body condition, immune response and reproductive traits were evaluated using mixed- model ANOVAs, with parental thermal condition as the fixed effects, and female identity and enclosure as the random effects. The effects of parental thermal treatment on hatching success was evaluated by generalized linear mixed models, with a logit link and the binomial family, female identity and enclosure as the random effects. The effects of parental and offspring thermal treatments (and their interaction) on growth rates and locomotor performance of offspring were evaluated by mixed-model ANOVAs, with parental and offspring thermal conditions as the fixed effects, and female identity and treatment enclosures of females and offspring as random effects. A stepwise cox regression analysis was used to detect the effect of parental and offspring thermal treatments on offspring survival.

## Results

### Thermal environments experienced by parents and offspring

We conducted the parental warming treatment experiments during the breeding season from April to August. Adults from the warming climate treatment experienced an average temperature of 25.93 ± 0.35 °C, which was 1.7 °C higher than that of the present climate treatment (24.26 ± 0.28 °C; *t = 25.90, df = 239, p < 0.001*). The offspring warming treatment experiment was initiated after hatching and continued until hibernation (from July to November). Offspring from the warming climate treatment experienced an average temperature of 24.33 ± 0.43 °C, which was 1.4 °C higher than that of the present climate treatment (22.96 ± 0.28 °C; *t = 16.02, df = 239, p < 0.001*; Fig. 1).

### Reproductive traits, hatching success, and incubation period

The parental thermal environment significantly affected oviposition date of the first clutch. Females from the warming climate treatment laid eggs 6 days (May 8th vs. May 14th) earlier on average than did females from the present climate treatment (*F*_1,37_ = 4.56, *P* = 0.04). However, parental thermal environment did not affect the majority of reproductive traits measured in our study, including clutch frequency, annual fecundity, total clutch mass, and reproductive output of each female (clutch size, clutch mass and egg mass; Table 1).

**Table 1:** Reproductive output of female *Takydromus septentrionalis* following different thermal treatments

Trait	Present climate	Warming climate	Mixed-model ANOVA
Clutch frequency	6.84 ± 0.48	7.00 ± 0.46	*F*_1, 2.00_ *=* 0.22*, P =* 0.90
Annual fecundity	20.35 ± 1.60	18.80 ± 1.55	*F*_1, 2.00_ *=* 0.33*, P =* 0.21
Total clutch mass (g)	6.49 ± 0.52	6.23 ± 0.51	*F*_1, 2.00_ *=* 0.06*, P =* 0.83
Clutch size	2.91 ± 0.08	2.68 ± 0.08	*F*_1, 2.04_ *=* 4.69*, P =* 0.16
Clutch mass (g)	0.93 ± 0.03	0.87 ± 0.03	*F*_1, 2.06_ *=* 4.05*, P =* 0.18
Egg mass (g)	0.33 ± 0.01	0.33 ± 0.01	*F*_1, 2.01_ *=* 0.06*, P =* 0.83

**Fig. 2:**
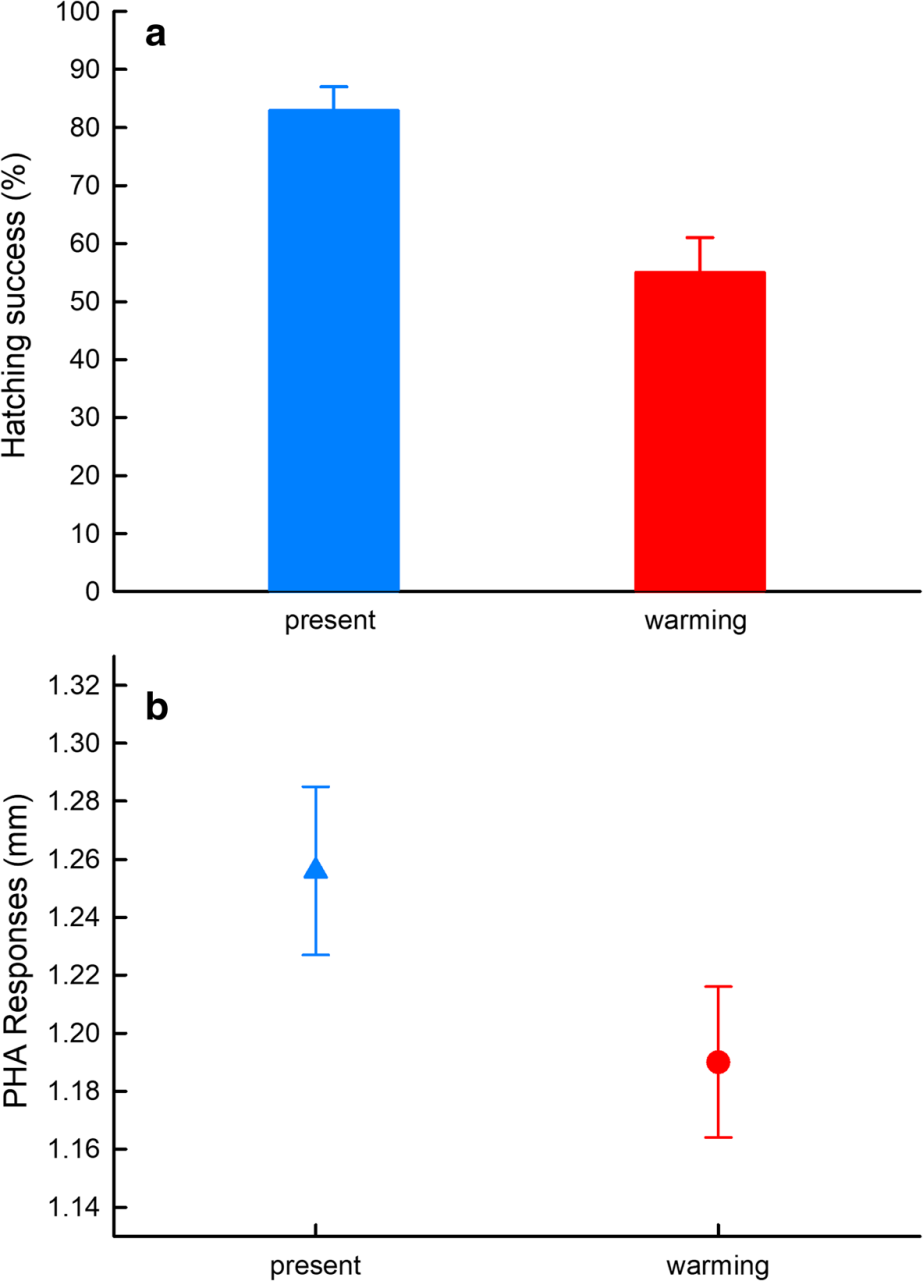
Hatching success of embryos (**a**) and female phytohemagglutinin (PHA) response (**b**) of *Takydromus septentrionalis* following exposure to different thermal treatments

Additionally, parental thermal treatment significantly affected hatching success of eggs (*t* = − 5.401, *df* = 345, *P* < 0.01), with lower hatching success for eggs from the warming climate treatment than for those from the present climate treatment (55 ± 6% vs. 83 ± 4%) (Fig. 2a). In contrast, parental thermal treatment did not significantly affect incubation period (45.62 ± 0.14 days for warming treatment vs. 46.07 ± 0.11 days for present climate treatment; *F*_1, 2.09_ = 3.17, *P* = 0.221).

### Body condition and cellular immune response of postpartum females

Parental thermal treatment did not influence the body condition of postpartum females (*F*_1,1.50_ = 9.113, *P* = 0.131), but significantly affected the PHA immune response of females (*F*_1,16_ = 9.765, *P* = 0.007), with a weaker cellular immune response in females from the warming climate compared to females from the present climate treatment (Fig. 2b).

### Body size, locomotion, growth, and survival of offspring

**Fig. 3:**
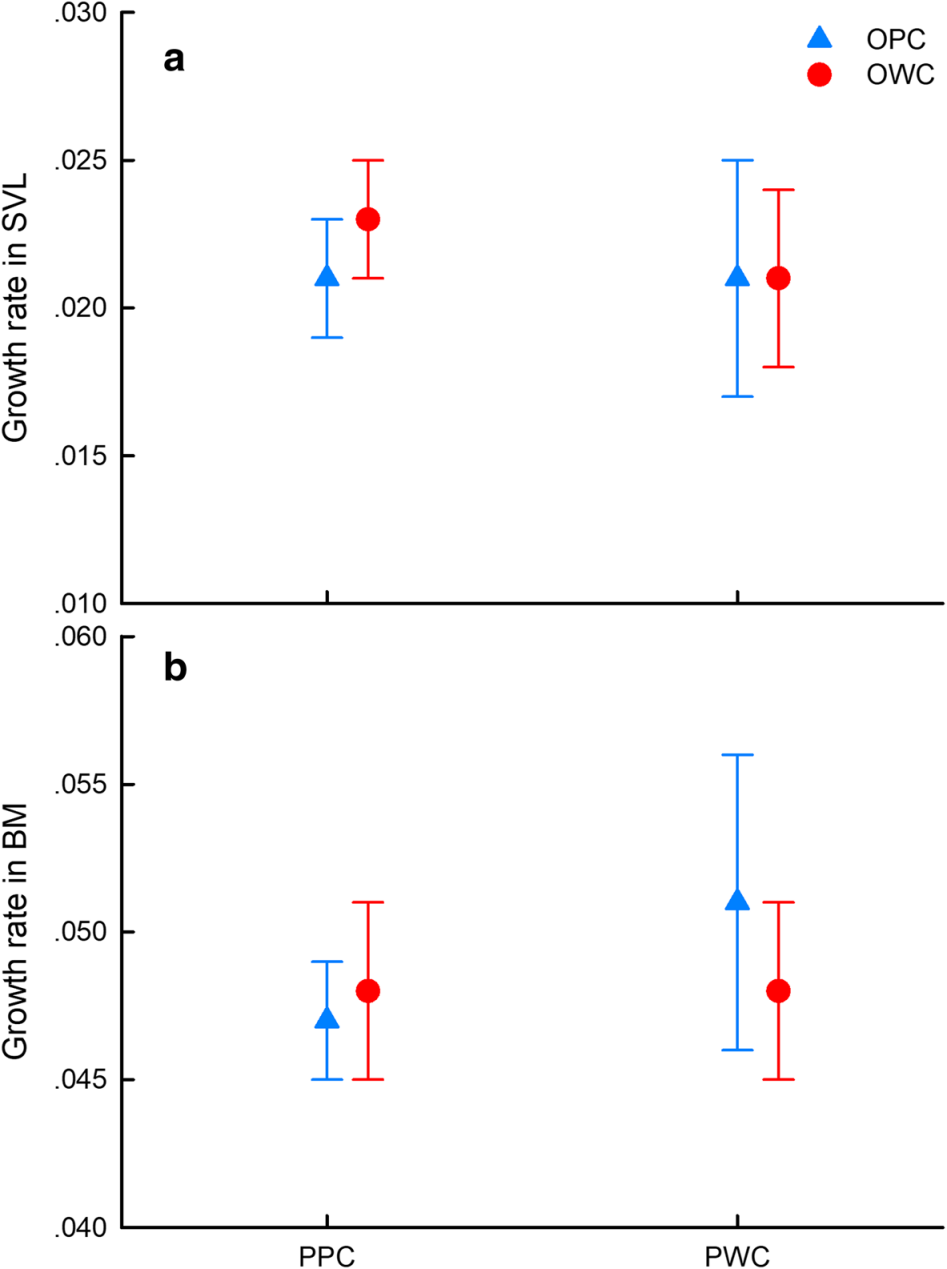
Maximum growth rates in snout-vent length (**a**) and body mass (**b**) of hatchling *Takydromus septentrionalis* following exposure to different maternal and offspring thermal treatments. PPC: Parental present climate, PWC: Parental warming climate, OPC: Offspring present climate, OWC: Offspring warming climate

**Fig. 4:**
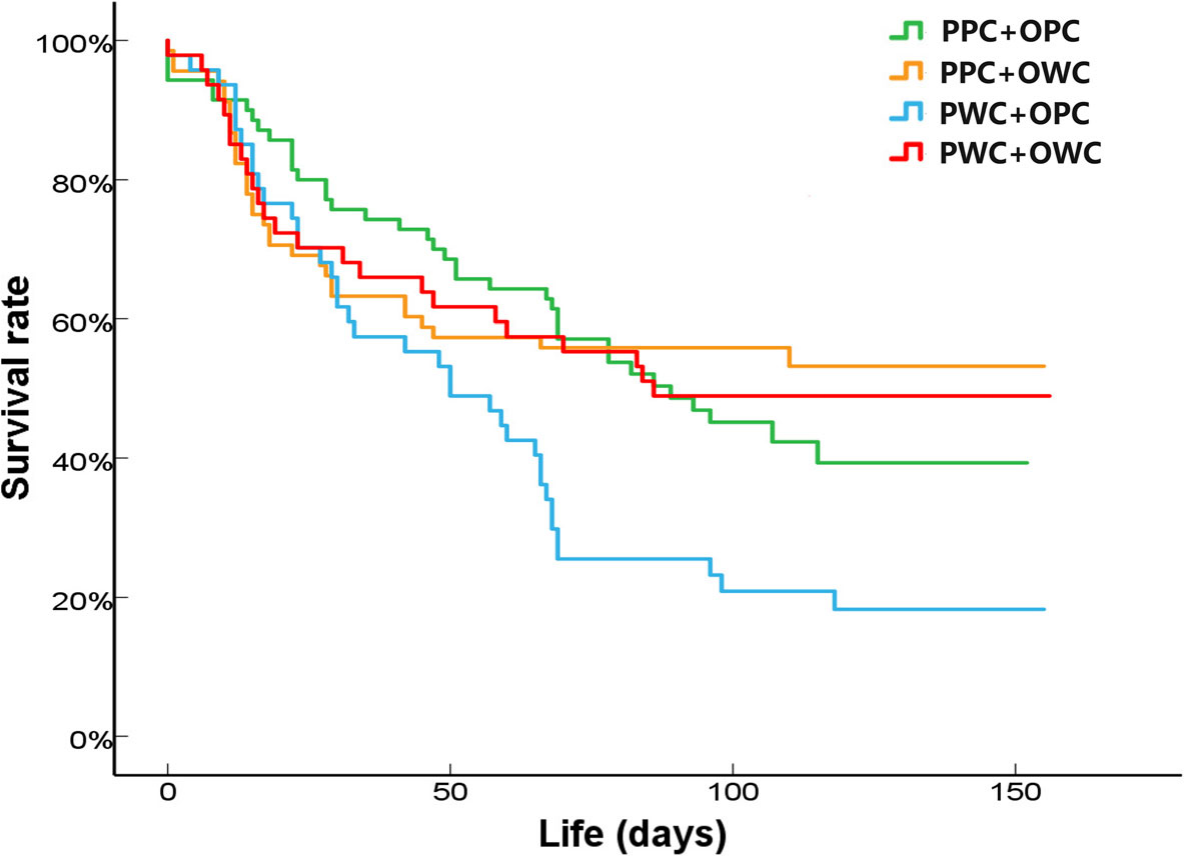
Survival rates of hatchling *Takydromus septentrionalis* following exposure to different maternal and offspring thermal treatments. PPC: Parental present climate, PWC: Parental warming climate, OPC: Offspring present climate, OWC: Offspring warming climate

Parental thermal treatment did not affect hatchling phenotypes including body size, body mass and locomotor performance (Table 2). Neither parental or offspring thermal treatment affected growth rate of hatchlings in terms of SVL (Parental: *F*_1,2.71_ = 0.12, *P* = 0.76; Offspring: *F*_1,18.46_ = 0.07, *P* = 0.80; Interaction: *F*_1,18.46_ = 0.21, *P* = 0.65) or body mass (Parental: *F*_1,3.82_ = 0.85, *P* = 0.41; Offspring: *F*_1,14.16_ = 0.04, *P* = 0.95; Interaction: *F*_1,14.16_ = 0.22, *P* = 0.65; Fig. 3). In contrast, parental thermal treatment significantly affected the survival of offspring, with lower survival rates for offspring from the parental warming climate treatment than for those from the present climate treatment (Wald χ^2^ = 10.474, *P* = 0.001; Fig. 4). However, parental and offspring thermal treatments had a significant interaction effect on offspring survival, with the lowest survival rates for offspring that experienced mismatched thermal environments (i.e., parental warming climate × offspring present climate) (Wald χ^2^ = 5.856, *P* = 0.016; Fig. 4).

**Table 2:** Body size and locomotion of hatchling *Takydromus septentrionalis* from females exposed to different maternal thermal treatments

Trait	Present climate	Warming climate	Mixed-model ANOVA
Snout-vent length (mm)	26.31 ± 0.11	26.66 ± 0.14	*F*_1, 2.02_ *=* 0.46*, P =* 0.56
Body mass (g)	0.39 ± 0.01	0.39 ± 0.01	*F*_1, 2.02_ *=* 0.01*, P =* 0.95
Average speed (cm/s)	56.45 ± 1.45	60.84 ± 1.79	*F*_1, 2.05_ *=* 1.06*, P =* 0.41
Sprint speed (cm/s)	69.14 ± 1.55	75.25 ± 1.92	*F*_1, 2.04_ *=* 1.64*, P =* 0.33

## Discussion

Although field enclosures provided relatively simple conditions compared to the complex environments that lizards experienced in nature habitats (e.g. food limitation and predation risk were eliminated), our study demonstrated that a simulated warming climate not only significantly affected female reproductive traits and egg hatchability, but also can, according to the scenario, have long-term effects on offspring survival in lizards. A warming climate advanced female reproductive phenology, but did not affect clutch size and offspring size. The low egg hatchability of females from the simulated warming climate indicated negative effects of climate warming on female reproduction in lizards. Moreover, offspring survival was higher when a matching environment was experienced by parents and offspring compared to when parents and offspring were exposed to a mismatching environment (namely the PWC × OPC treatments, but not the PPC × OWC treatments), partially giving support to the environmental matching hypothesis. Below we explore the potential causes and the ecological significance of these warming effects on lizard life history.

Compared to present climate conditions, a simulated warming climate significantly advanced the timing of reproduction (oviposition), which is a well-known phenomenon in the animal kingdom [4]. The advanced timing of reproduction is largely due to rapid accumulation of energy for reproduction and endocrine control of reproductive cycling induced by temperature increase [53, 63]. However, as found in some other lizard species [41, 64, 65], a climate warming scenario did not affect clutch size or egg mass in the northern grass lizard, *T. septentrionalis*. Several reasons may account for the insensitivity of female reproductive output to climate warming in the northern grass lizard. First, active behavioral thermoregulation by female northern grass lizards can partially reduce the body temperature differential between lizards from a warming treatment and their conspecifics exposed to present climate conditions [58]. Similar use of behavioral thermoregulation to alleviate the effects of a warming climate has been shown in several other oviparous lizards (*Scincella modesta* and *Amphibolurus muricatus*) [18, 65]. Second, unlike some insects that can adjust the relationship between egg size and number on the basis of food quality [66, 67], female *T. septentrionalis* likely allocate optimal energy to a clutch of eggs and developing offspring, resulting in relatively fixed egg and clutch sizes that are resistant to environmental perturbations such as thermal heterogeneity and food availability [19, 58, 68]. Nonetheless, a substantial increase in environmental temperatures may increase clutch frequency in *T. septentrionalis* [19] or clutch size in some other species [21, 41, 69]. Third, the low immune capability (Fig. 2b) of females from the warming climate treatment might be due to the high temperatures that directly depress the immunity, or induced by decreased energetic allocation to immunity, as a result of resource-allocation trade-offs between immunity and reproduction (e.g. [70, 71]). Additionally, as the terminal investment hypothesis predicts, expending a greater amount of energy resources on current reproduction rather than maintenance (and therefore potential future reproduction) is a strategy that would have selective advantages when animals perceive there to be a higher probability of mortality and lower chances of reproducing in the future [72–74].

Our experiments indicated that when parents are exposed to a warming environment, embryo viability is significantly decreased, as shown by the low hatching success of eggs from these parents. This provides unequivocal evidence for the assertion that embryos are a fragile life history stage that might be extremely sensitive to climate change [75, 76]. The low viability of embryos could be directly attributable to the thermal environment experienced by embryos in utero, as embryos may be very sensitive to temperature at earlier developmental stages (e.g., cell differentiation and organ formation) (e.g., [77, 78]). Alternatively, temperature change may affect maternal investment (e.g., hormones, nutrition or immunity proteins) into an egg that may affect egg hatchability later [79].

Interestingly, we found that offspring from parents who experienced a warming environment survived well under a simulated warming climate, but not under a present climate. This is consistent with the prediction of the environmental matching hypothesis which suggests that the parental effect would be adaptive when the developmental conditions match post-developmental conditions, but detrimental when these conditions are mismatched [25, 80]. Nonetheless, the detrimental effect in our case was evident only when offspring from the parental warming climate treatment experienced present climate later, not when offspring from the parental present climate treatment experienced warming climate later. Parents that experience a warming climate anticipate that the offspring will also live in a warming environment, and thus ‘program’ their offspring phenotype accordingly, to boost offspring survival (as predicted by the anticipatory parental effects) [25, 81, 82]. Similarly, parental thermal environment affects offspring growth in a fish, with higher growth rates in water temperatures matching those of their parents [40]. The ecological and evolutionary significance of this finding depends on the links between parental and offspring thermal conditions under climate change. In China, ambient temperatures are predicted to increase substantially in winter, spring, and autumn, but not in summer [83]. Under this predicted climate scenario, *T. septentrionalis* parents will experience a warming spring, but their hatchlings emerging in early summer will experience an unwarmed summer, and thus, the mismatch between parental (warm) and offspring (present) thermal environments may decrease offspring survival. In contrast, survival rates of hatchlings emerging in late summer would not be expected to decrease, because a warming autumn may enhance offspring survival despite gravid females experiencing a normal summer. Accordingly, the fitness consequences of anticipatory parental effects largely depend on seasonal variation in climate warming. Global change will increase temporal variation in climatic conditions [59], which could favor plasticity [30, 84], but also complicate the temporal matching between climate conditions and anticipatory parental effects. This makes any predictions about organismal fitness more complicated, and thus, further exploration on this subject would be of great interest to fully understand the impact of climate warming on organisms.

## Conclusion

Overall, our findings of negative effects of warming on female immunity and embryonic development, and the complex interaction between parental and offspring environments on offspring survival highlight the importance of taking multiple life-history stages into account when we evaluate the impact of climate warming on the fitness of a given species. In addition, our study demonstrates anticipatory parental effects in response to a warming climate in an ectothermic vertebrate, yet the final fitness consequences of these parental effects depend on future climate change scenarios. Although a growing body of evidence suggests that transgenerational plasticity plays an important role in species adaptation in thermally variable environments and may mediate impacts of climate change on both plants and animals [26, 31, 38, 39, 85], our results highlight that transgenerational effects could buffer the adaptive potential of ectothermic animals in thermally variable environments.

## Additional files

Additional file 1: Ambient temperatures of sampling site and enclosure site, and the active body temperatures of lizards in enclosures. (DOCX 195 kb)

Additional file 2: Data used in this study. (XLSX 90 kb)

## Abbreviations

OPC: Offspring present climate
OWC: Offspring warming climate
PHA: phytohemagglutinin
PPC: Parental present climate
PWC: Parental warming climate
SVL: Snout-vent length
