# Supplementary material for: Anticipatory parental effects in a subtropical lizard in response to experimental warming
Source: Front Zool. 2018 Dec 5;15:51. doi: 10.1186/s12983-018-0296-3 (PMC6282349; doi:10.1186/s12983-018-0296-3)
Supplement: Supplementary file 1 — Ambient temperatures of sampling site and enclosure site, and the active body temperatures of lizards in enclosures. (DOCX 195 kb) [file 12983_2018_296_MOESM1_ESM.docx]

**Fig.S1 Ambient temperature of Zhoushan and Hangzhou where we collected *Takydromus septentrionalis* lizards and built enclosures, respectively.**

We calculate mean daily temperatures on the basis of temperature records from 1980 to 2012 in the two sites of Zhoushan and Hangzhou, eastern China ([http://www.nmic.gov.cn)](http://www.nmic.gov.cn).). Mean daily temperatures are very similar in the two sites during the breeding season (April-November) (*F*_1, 490_ =0.063, *p* = 0.803), and the entire year as well (*F*_1, 728_ =0.005, *p* = 0.944).





**Fig. S2 Active body temperatures of *Takydromus septentrionalis* lizards in early May.**

Active body temperatures of lizards in present climate treatment (Blue triangles) and simulated warming climate treatment (Red circles) were measured on sunny day of May 1^st^ by an infrared thermometer at 9:00, 12:00, 15:00, 18:00 O’clock, respectively. The mean daily body temperature was 29.16 ± 0.31℃ or 31.43 ± 0.40℃ for lizards in the present climate treatment or the simulated warming climate treatment.
